# Molecular Cytogenetic and Agronomic Characterization of the Similarities and Differences Between Wheat–*Leymus mollis* Trin. and Wheat–*Psathyrostachys huashanica* Keng 3Ns (3D) Substitution Lines

**DOI:** 10.3389/fpls.2021.644896

**Published:** 2021-03-31

**Authors:** Jiachuang Li, Jiaojiao Li, Xueni Cheng, Li Zhao, Zujun Yang, Jun Wu, Qunhui Yang, Xinhong Chen, Jixin Zhao

**Affiliations:** ^1^Shaanxi Key Laboratory of Plant Genetic Engineering Breeding, College of Agronomy, Northwest A&F University, Xianyang, China; ^2^College of Life Sciences, Northwest A&F University, Xianyang, China; ^3^School of Life Sciences and Technology, University of Electronic Science and Technology of China, Chengdu, China; ^4^College of Agronomy, Northwest A&F University, Xianyang, China

**Keywords:** wheat, *Psathyrostachys huashanica*, *Leymus mollis*, Ns genome chromosome, substitution lines

## Abstract

*Psathyrostachys huashanica* Keng (2*n* = 2*x* = 14, NsNs) and *Leymus mollis* Trin. (2*n* = 4*x* = 28, NsNsXmXm) are valuable resources for wheat breeding improvement as they share the Ns genome, which contains diverse resistance genes. To explore the behaviors and traits of Ns chromosomes from the two species in wheat background, a series of wheat–*P. huashanica* and wheat–*L. mollis* substitution lines were developed. In the present study, line DH109 (F_7_ progeny of wheat–*P. huashanica* heptaploid line H8911 × durum wheat Trs-372) and line DM131 (F_8_ progeny of wheat–*L. mollis* octoploid line M842 × durum wheat Trs-372) were selected. Cytological observation combined with genomic *in situ* hybridization experiments showed that DH109 and DM131 each had 20 pairs of wheat chromosomes plus a pair of alien chromosomes (Ns chromosome), and the pair of alien chromosomes showed stable inheritance. Multiple molecular markers and wheat 55K SNP array demonstrated that a pair of wheat 3D chromosome in DH109 and in DM131 was substituted by a pair of *P. huashanica* 3Ns chromosome and a pair of *L. mollis* 3Ns chromosome, respectively. Fluorescence *in situ* hybridization (FISH) analysis confirmed that wheat 3D chromosomes were absent from DH109 and DM131, and chromosomal FISH karyotypes of wheat 3D, *P. huashanica* 3Ns, and *L. mollis* 3Ns were different. Moreover, the two lines had many differences in agronomic traits. Comparing with their wheat parents, DH109 expressed superior resistance to powdery mildew and fusarium head blight, whereas DM131 had powdery mildew resistance, longer spike, and more tiller number. Therefore, Ns genome from *P. huashanica* and *L. mollis* might have some different effects. The two novel wheat–alien substitution lines provide new ideas and resources for disease resistance and high-yield breeding on further utilization of 3Ns chromosomes of *P. huashanica* or *L. mollis*.

## Introduction

As one of the three major cereals in the world, common wheat (*Triticum aestivum* L., 2*n* = 6*x* = 42, AABBDD) makes tremendous contributions to the development of human civilizations. The origin of common wheat was the result of natural distant hybridization between *Triticum* and *Aegilops* (Chantret et al., 2005). It is widely believed that wild relative species of wheat possess numerous excellent traits, i.e., disease resistance, stress tolerance, and high productivity, which are what common wheat needs. Because Barelle did the first artificial interspecific hybridization in wheat in the early 19th century and found that offspring had improved adaption to different environments (Ciferri, 1955), interspecific and intergeneric hybridization of common wheat were always important directions for breeders. So far, nearly 90 species from 14 relative genera of wheat have successfully crossed with common wheat, and a series of wheat-related species germplasm resources with outstanding agronomic traits have been developed (Pauk, 2016). For example, wheat–*Secale cereale* 1BL/1RS translocation, 1B (R) substitution, and 4R addition lines had high productivity and resistance to stripe rust (Rabinovich, 1998; An et al., 2013); wheat–*Haynaldia villosa* 6VS/6AL and 4VS/4DL translocation lines conferred resistance to wheat powdery mildew and streak mosaic virus, respectively (Chen et al., 1995; Zhang et al., 2005); wheat–*Thinopyrum ponticum* 7J^st^ (7B) and 1st (1B) + 4St-4J^st^ (4B) substitution lines exhibited resistance to wheat stripe rust (Li et al., 2015; Zhu et al., 2017); and wheat–*Agropyron cristatum* 6P addition and 2P translocation lines had high resistance to wheat powdery mildew (Han et al., 2014; Jiang et al., 2018).

*Psathyrostachys huashanica* Keng (2*n* = 2*x* = 14, NsNs) belongs to *Psathyrostachys* Nevski, which is diploid perennial plant containing approximately 10 species possessing Ns genome (Baden, 1991; Wang et al., 1994). *P. huashanica* occurred only on the stony slopes of Huashan Mountains, Shaanxi Province, China, and owned numerous excellent traits, such as resistance to wheat disease (rust, take-all, scab, and powdery mildew), tolerance to abiotic stress (salinity, alkalinity, and cold), and early maturation (Baden, 1991; Jing et al., 1999; Wang and Shang, 2000; Song et al., 2013). The first distant hybridization between common wheat and *P. huashanica* was conducted successfully in our laboratory by Chen (1991) using embryos culture method. The F_1_ hybrid H811 (2*n* = 6*x* = 28, ABDNs) as female parent backcrossed with common wheat line 7,182 to generate heptaploid hybrid H8911 (2*n* = 7*x* = 49, AABBDDNs), which was used to self-cross or cross with other wheat cultivars to develop several wheat–*P. huashanica*–derived lines.

*Leymus mollis* Trin. (2*n* = 4*x* = 28, NsNsXmXm) is a cross-pollinated heterotetraploid perennial species of *Leymus* Hochst., and it grows mostly on the coastal beaches. *L. mollis* was regarded as a suitable exogenous germplasm for wheat improvement because of its outstanding traits including long spikes, full-spikelet, strong stems, immunity to diseases caused by bacteria and fungi, and high tolerance to saline–alkali soil (Mujeeb-Kazi and Rodriguez, 1981; Kishii et al., 2003). Distant hybridization between *Leymus* and common wheat could date back to late 1960s (Tsitsin, 1965). Later, the wheat–*L. mollis* octoploid derivative line M842–12 (2*n* = 8*x* = 56, AABBDDNsNs) and M842–13 (2*n* = 8*x* = 56, AABBDDXmXm) were created by Fu et al. (1993) via embryo rescue and colchicine treatment. Subsequently, a series of wheat–*L. mollis–*derived germplasms were obtained mainly by using octoploid *Tritileymus* M842-12 to cross with wheat cultivars.

The original studies suggested that the genome of *Leymus* was from two genera: *Thinopyrum* Löve (E genome) and *Psathyrostachys* Nevski (Ns genome) based on its rhizomatous growth habit and saline habitat (Dewey, 1984; Löve, 1984). The existence of Ns genome has been verified using cytogenetic molecular methods (Wang and Hsiao, 1984; Sun et al., 1995). But, Zhang and Dvořák (1991) raised question about the presence of E genome, and they were supported by subsequent studies from other researchers (Ørgaard and Heslop-Harrison, 1994; Wang and Jensen, 1994). As the other genome was unknown, the genome of *Leymus* was assigned as NsNsXmXm (Xm meaning the unknown genome) (Wang et al., 1994). However, up to now, which species of *Psathyrostachys* Nevski donates the Ns genome is still unclear.

In the present study, two novel wheat-alien–derived lines were developed to judge whether *P. huashanica* was a donator of Ns genome to *Leymus* and whether Ns genome chromosomes from the same homoeology but different genera would express the same agronomic traits in wheat background. The objectives of the research were to (a) develop wheat–*P. huashanica* and wheat–*L. mollis* substitution lines, (b) identify inherent stability and homoeologous group of alien chromosomes in wheat background, and (c) investigate agronomic and morphologic traits of two lines.

## Materials and Methods

### Development of Plant Materials

The plant materials used in this study included *P. huashanica* Keng (2*n* = 14, NsNs), *L. mollis* pilger (2*n* = 28, NsNsXmXm), common wheat (2*n* = 42, AABBDD) lines 7,182, Mingxian169 (MX169), Chinese Spring (CS) and Huixianhong (HXH), *Triticum* durum (2*n* = 28, AABB) line Trs-372, wheat–*P. huashanica* disomic substitution line DH109, and wheat–*L. mollis* disomic substitution line DM131. DH109 was obtained from the F_7_ progeny of wheat–*P. huashanica* heptaploid line H8911 × line Trs-372. The hexaploid hybrid H8911 (2*n* = 49, AABBDDNs) was generated from the cross between common wheat line 7,182 and *P. huashanica.* DM131 was obtained from the F_8_ progeny of wheat–*L. mollis* octoploid line M842 × line Trs-372. The octoploid hybrid M842 (2*n* = 56, AABBDDNsNs) was generated from the cross between common wheat line 7,182 and *L. mollis*. MX169, CS, and HXH were susceptible controls in the disease resistance testing. All materials were deposited at the College of Agronomy, Northwest A&F University, China. Total genomic DNA was extracted using the standard CTAB method.

### Cytological Observation

The roots and young spikes were sampled at appropriate stages, when the lengths of roots and panicles were 1–2 and 5–6 cm, respectively. Samples were pretreated in an ice–water bath for 24 h before transfer to Carnoy’s fixative fluid I (ethanol: glacial acetic acid mixture at 3:1, vol/vol) for 24 h and finally to 70% ethanol and stored at −20°C. After treatment with 1% cellulase (Yakult, Japan) and 2% pectinase (Yakult, Japan) at 37°C for 1 h, the root tips were cleaved into signal cell to facilitate the observation of chromosomal number and morphology. Anthers were taken from the middle to both sides of the spike until target stages and the microsporocytes were stained with 1% acetocarmine before cytological observations. The slides with good split phases were dried and marked for the following experiments. These slides’ preparation for fluorescence *in situ* hybridization (FISH) and genomic *in situ* hybridization (GISH) analysis was through UV crosslinking (1,250 mj/cm^2^, 3 min) which make chromosomes attached on slides. In this process, the root and spikes were numbered to ensure derivation from the same one seed. Twelve plants of each line were randomly selected for cytological screen and *in situ* hybridization analysis for 5 consecutive years.

### GISH Analysis

In GISH experiment, genomic DNA from alien donor *P. huashanica* and *L. mollis* labeled with DIG-11-dUTP were used as probes for GISH analysis. After more than 16-h hybridization in a dark and moist box at 37°C, anti-digoxigenin fluorescein kit (Roche, Germany) was used to visualize the combinative zone of probes, and Vectashield H-1300 (VECTOR, United States) was used to counterstain the chromosomes. Detailed procedures could be seen in an article by Zhao et al. (2013). Fluorescent signals were observed with a microscope (ZEISS Imager M2, Germany) and imaged (ZEISS ICc5, Germany).

### DNA Marker Analysis

Two hundred six pairs of simple sequence repeat (SSR) primers from each wheat chromosome were selected to determine the chromosomal composition of DH109 and DM131. SSR markers included GWM series developed by Röder et al. (1998), GDM series developed by Pestsova et al. (2000), and CFA and CFD series developed by Sourdille et al. (2004). One hundred twenty-four pairs of expressed sequence tag–sequence tag site (EST-STS) primers^1^ distributed among seven wheat homoeologous groups with corresponding chromosomes of Ns genome were employed to determine the homoeology of the introduced alien chromosomes in two lines. In addition, nine pairs of *P. huashanica* Ns genome–specific sequence characterized amplified region (SCAR) markers (Chen et al., 2010; Wang et al., 2014; Su et al., 2015) located in the 1Ns, 3Ns, and 5Ns chromosomes were used for additional chromosomes verification.

The products of EST-STS and SSR markers experiments were electrophoresed on 8% non-denaturing polyacrylamide gel (constant voltage 165 V, 2.5 h) and stained with alkaline-silver method. The products of SCAR markers were separated on 1% agarose gels (150 V, 0.5 h) and observed using BIO-RAD chemiDoc XRS + (ImageLab system, United States). The specific and parallel bands amplified from the two lines and alien parents by EST-STS markers were sequenced by Sangon, China, and aligned using DNAMAN V6.0.3 (Lynnon Biosoft, United States) and BLAST tool on NCBI^2^ and URGI^3^.

### FISH and Sequential GISH

In FISH experiment, a pair of fluorescent-modified probes comprising oligo-pSc119.2 (6-FAM-5′) and oligo-pTa535-1 (TAMRA-5′) (Danilova et al., 2012; Tang et al., 2014) was used to identify the chromosomal compositions of DH109 and DM131. Homoeologous group of each wheat chromosome could be distinguished according to fluorescent spots spread on chromosomal arm. The chromosomal FISH signals of the two materials were compared with karyotype of Chinese Spring and Mianyang 11 provided by Tang et al. (2014). Oligo-primers were dissolved in 1 × TE solution to 20 ng/μL. Each slide with mixture (3 μL pSc119.2, 2 μL pTa535-1, and 5 μL 1 × TE) was put in a moisturizing black box at 55°C for more than 3 h. Then, slides were immersed in a 2 × SSC solution to make coverslip slip. Vectashield H-1200 (VECTOR, United States) was used to counterstain the chromosomes. For the sequential GISH, the slides photographed were soaked in 75% alcohol for 5 min and exposed to light for 24 h. The protocol of sequential GISH was the same as provided in GISH analysis. Fluorescent signals were observed with a microscope (ZEISS Imager M2, Germany) and imaged (ZEISS ICc5, Germany).

### Wheat 55K SNP Array Analysis

Purifying genomic DNA of materials was hybridized to wheat 55K SNP genotyping arrays, and Illumina Bead Array technology was used for scanning in China Golden Marker Biotechnology Company (Beijing, China). The wheat 55K SNP array contained 49,078 SNPs, which were distributed across 21 pairs of wheat chromosomes. The total valid number of markers divided by the marker number that had the same genotype in a chromosome between two lines was calculated as the percentage of the same markers on each chromosome. Excel 2016 (Microsoft, United States) was used for statistics and analysis of data. Row picture and chromosome map used SigmaPlot V12.5 (SYSTAT software, Inc., United States) and MapChart V2.32 (Wageningen University & Research, Netherlands), respectively.

### Evaluation of Disease Resistance and Agronomic Traits of Materials

Resistance to wheat common diseases [stripe rust, powdery mildew, and fusarium head blight (FHB)] and agronomic traits of materials were evaluated for 3 consecutive years (2018–2020) in Yangling, China. In the field condition, the materials were arranged separately in a completely randomized block design, and each material had two rows with 12-cm interval of each plant. In the incubator, all materials were separated planted in one plug, and the controls were in the center and four corners for better infection. Meanwhile, five plants of each material were arranged with three replications. For the evaluation of disease resistance, 12 plants of each material were investigated in the same way every year. For the evaluation of agronomic traits, the average data based on five samples and three repeats of every year to ensure that accurate results were obtained.

Six morphological traits comprising the plant height, tiller number, spike length, spikelet number, kernel number, and thousand-kernel weight were investigated. Grain quality indicators of materials, including the kernel protein content, gluten protein content, starch content, subsidence value, volume weight, dough stability time, and flour field, were tested by Perten DA 7250 NIR analyzer (Sweden). Significant analyses between different materials were conducted using the SPSS Statistics 20 software program (IBM Corp., Armonk, NY, United States).

To access the adult plant resistance to stripe rust of two lines, three different races of *Puccinia striiformis* f. sp. *tritici* (CYR32, 33, 34) were used for artificial inoculation at an appropriate period. Mixed races were smeared onto the wheat flag leaves of every experimental material after a drizzle of early spring for better infection. The susceptible cultivar was Mingxian 169, and the infection types (ITs) to stripe rust were graded according to the method provided by Ma et al. (1995). IT was rated on a scale from 0 to 4, in which 0 and 0 indicate immune and nearly immune, 1 and 2 denote high resistance and moderate resistance, and 3 and 4 indicate moderately susceptible and susceptible, respectively. Each type can be appended with “+” or “−” to indicate that it is heavier or lighter.

The evaluation of resistance to powdery mildew was conducted at the seeding stage in a growth chamber. The *Blumeria graminis* f. sp. *tritici* isolate E09 was used for inoculation when materials were at two-leaf stage, and Huixianhong was susceptible control. Pathogen spores with high activity were dusted onto the leaves, and plants were incubated at 22°C and 70% humidity for 15 days. The ITs to powdery mildew were scored using the method of Sheng (1988) on five grades, which were IT = 0 and 0 indicating immune and nearly immune, IT = 1 and 2 denoting high resistance and moderate resistance, and 3 and 4 meaning moderately susceptible and susceptible, respectively. Each type can be appended with “+” or “−” to indicate that it is heavier or lighter.

The type II resistance to FHB was evaluated at field using the method of single floret inoculation described by Bai et al. (1999; 2000). In brief, 10 randomly selected spikes at flowering period were injected 10 μL of conidial spore suspension into the floral cavity between the lemma and palea of a single floret in the middle of one spike. *Fusarium graminearum* Schwabe strain PH1 was expanded and diluted to 100 spores μL^–1^ in mung beans liquid medium. Each inoculated spike was covered with a moist plastic bag for 2 days, and total spikelets and infected spikelets were counted at 21 days after injection (Bai, 1996). The infected grades basing on symptomatic spikelet of entire spike were 1 to 5, where 1 indicates no extension to cob and 5 means symptomatic spikelet more than three-fourths of the whole spike. Intermediate infection grades of spike were represented by 2 (less than 1/4), 3 (1/4 to 1/2), and 4 (1/2 to 3/4). Reaction index (RI, 1–5) and infected spikelet rate (ISR,%) of materials to FHB were according to Yang et al. (1998) as follows: RI = Σ (number of spikes at each infected grade × correspond infected grade)/number of total spikes, where RI = 1.1–2.0 denotes resistance, 2.1–3.0 denotes moderate resistance, 3.1–4.0 denotes moderately susceptible, and 4.1–5.0 denotes susceptible); ISR = Σ (infected spikelets/total spikelets)/number of total spikes.

## Results

### Observation of Cytogenetics of DH109 and DM131

**FIGURE 1:**
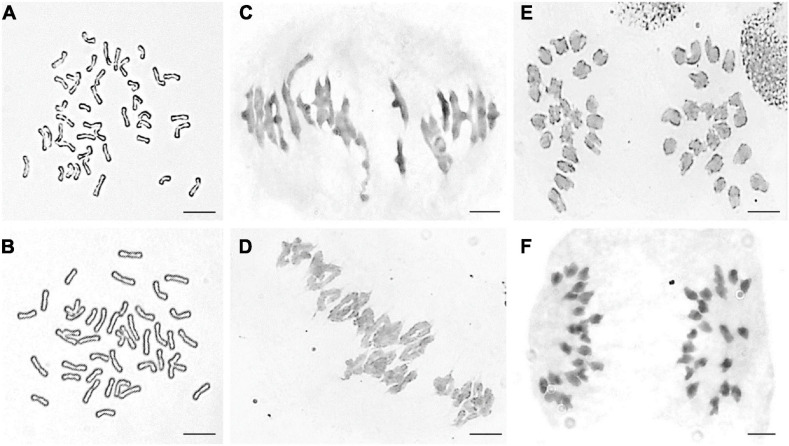
Cytological observation of root tip cells (RTCs) and pollen mother cells (PMCs). **(A)** Mitotic metaphase of DH109, 2*n* = 42. **(B)** Mitotic metaphase of DM131, 2*n* = 42. **(C)** Meiotic metaphase I of DH109, 2*n* = 21 II. **(D)** Meiotic metaphase I of DM131, 2*n* = 21 II. **(E)** Meiotic anaphase I of DH109, 2*n* = 21 + 21. **(F)** Meiotic anaphase I of DM131, 2*n* = 21 + 21. Scale bar, 10 μm.

Part division phases of root tip cells (RTCs) and pollen mother cells (PMCs) were observed to clarify chromosomal numbers and pairing. The mitosis metaphase observations indicated that RTCs of line DH109 (Figure 1A) and line DM131 (Figure 1B) both had a chromosome number of 42. PMCs in meiotic metaphase I showed that the DH109 had a chromosome configuration of 21 bivalents without a trivalent or quadrivalent (Figure 1C) and so had DM131 (Figure 1D). Meiotic anaphase I of PMC could exhibit segregation of homoeologous chromosomes. In PMCs of line DH109 (Figure 1E) and line DM131 (Figure 1F), chromosomes segregated and moved to the cell poles normally at meiotic anaphase I. These results indicated that DH109 and DM131 were cytological stable lines with regular chromosome number and cell division.

### GISH Analyses of DH109 and DM131

**FIGURE 2:**
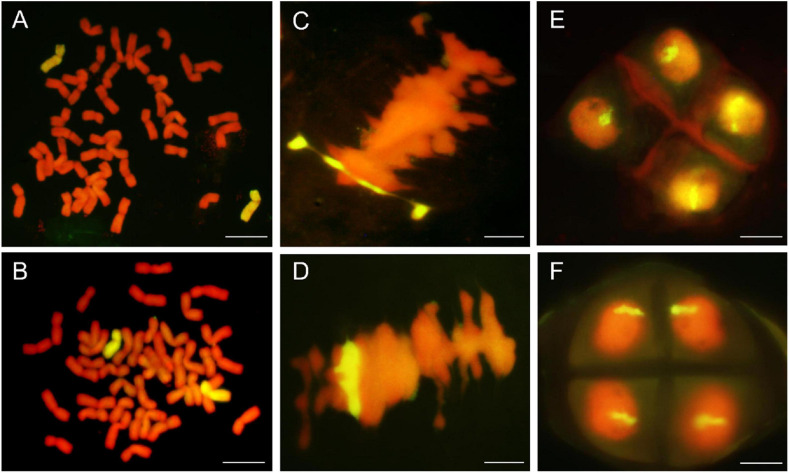
Genomic *in situ* hybridization (GISH) analysis of line DH109 and line DM131. GISH analysis of RTCs in the mitotic metaphase. **(A)** In DH109, two chromosomes with yellow–green signals were detected as alien chromosomes from *P. huashanica*. **(B)** In DM131, two chromosomes with yellow–green signals were detected as alien chromosomes from *L. mollis*. GISH analysis of PMCs in the mitotic metaphase. **(C)** Alien chromosomes formed a rod bivalent with fluorescent signal in DH109. **(D)** Alien chromosomes formed a ring bivalent with fluorescent signal in DM131. GISH analysis of gametes in the meiosis telophase II. **(E)** Each of the four progeny cells had a fluorescent signal in DH109. **(F)** Each of the four progeny cells had a fluorescent signal in DM131. Chromosomes were counterstained with propidium iodide (red). Scale bar, 10 μm.

Genomic *in situ* hybridization analyses were using the whole genomic DNA from *P. huashanica* for DH109 and *L. mollis* for DM131 as the probe and common wheat parent as the block to identify number of alien chromosome in the derived line. The results of RTCs GISH analysis showed that line DH109 had two chromosomes from *P. huashanica* (Figure 2A), and line DM131 had two chromosomes from *L. mollis* (Figure 2B). GISH analysis of PMCs in meiotic metaphase I showed that a rod bivalent with yellow–green signals in DH109 (Figure 2C) and a ring bivalent with hybridization signal in DM131 (Figure 2D). In meiotic telophase II, each of the four sperms carried an alien chromosome in DH109 and DM131 according to Figures 2E,F, respectively. Mitotic correlative results demonstrated that the two lines were both disomic substitution lines in which two wheat chromosomes were substituted by Ns chromosomes, and the alien chromosomes in DH109 were from *P. huashanica* and were from *L. mollis* in DM131. PMCs’ GISH analysis indicated that the two substitution lines were cytogenetically stable wheat-alien–derived lines for the alien chromosomes could pair, segregate, and inherit normally.

### Multiple Molecular Markers Analysis of DH109 and DM131

**FIGURE 3:**
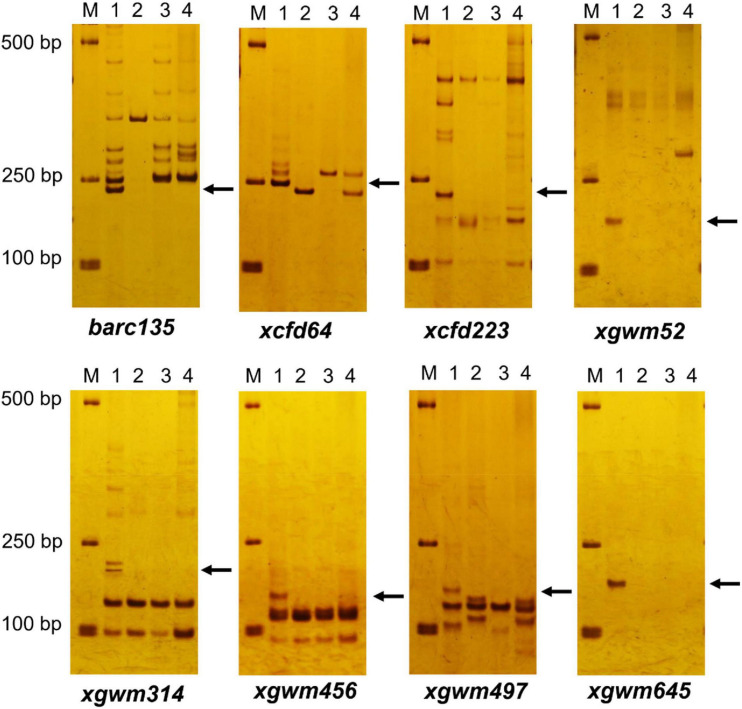
Simple sequence repeat (SSR) markers analysis to genotypes DH109 and DM131. Among 206 pairs of SSR markers, eight pairs of markers comprising *barc135*, *xcfd64*, *xcfd223*, *xgwm52*, *xgwm314*, *xgwm456*, *xgwm497*, and *xgwm645* on the wheat 3D chromosomes did not amplify 3D genome–specific bands in *durum* wheat Trs-372, DH109, and DM131. Lane M, DL2000 marker; lane 1, common wheat 7182; lane 2, Trs-372; lane 3, DH109; lane 4, DM131. Arrows indicate the missing D genome–specific bands.

Among the 206 pairs of SSR markers spread on 21 pairs of wheat chromosomes, eight pairs of markers, including *barc135*, *xcfd64*, *cxfd223*, *xgwm52*, *xgwm314*, *xgwm456*, *xgwm497*, and *xgwm645* related to wheat 3D chromosomes could amplify wheat D genome–specific bands in common wheat 7,182, but not in durum wheat Trs-372 and two derived lines (Figure 3 and Table 1). The results indicated that both DH109 and DM131 lost their wheat 3D chromosomes.

**TABLE 1:** Molecular markers and FISH oligo-primers used in this study to analyze the chromosomal composition of DH109 and DM131.

Marker	Type	Primer (5′–3′)	Tm (°C)	Location
BE605103	STS	**F:** ACCGACATCACCCATGTCTT, **R:** CGGCATAGACGGATAGGCT	60	3AL 3BL 3DL
BE637806	STS	**F:** TCGCAGATCTTCGTTGTTTG, **R:** GGGAATGTGTGGATATTCGG	60	3AS 3BS 3DS
BF291730	STS	**F:** TTAAGAACCCAACCCACAGC, **R:** AGCAGCGCACGGTATTTACT	60	3AL 3B 3DL
CD452402	STS	**F:** ACATACACCCTCTTGCCGTC, **R:** GCTTCTTCAAAAGGGCAGTG	60	3AL 3BL 3DL
CD454575	STS	**F:** AAGGGGTACCCGCATAATTC, **R:** TCTGAGATACCAGGGATGGC	60	3BS 3DS
BF200774	STS	**F:** AGTTCTTCAGCGTGTGCCTT, **R:** AATGTGGTGTTCATGGGGAT	60	3AL 3BL 3DL
BF429203	STS	**F:** CTTCGTAGCCTCCTCACTGG, **R:** AGATTATGTGCGTGCTGTGC	60	3AL 3BL 3DL
BM137713	STS	**F:** CTGTCCTTGTAATGGTCCCTG, **R:** AGGTAAAAGCCGGTTCGGT	60	3AL 3BL 3DL
BG263365	STS	**F:** AACTATCGATGAGATGCGGG, **R:** GAAGCCTTGGAGACCTCCTT	60	3AL 3BL 3DL
CD454086	STS	**F:** CTCTGTGTGTGGCACTCCAT, **R:** ATTGCTCGAGATGATGGGTC	60	3AL 3BL 3DL
barc135	SSR	**F:** ATCGCCATCTCCTCTACCA, **R:** GCGAACCCATGTGCTAAGT	52	3DS
xcfd64	SSR	**F:** ACAGTGTTGTTGCCCCTTTC, **R:** CCCATGTTACAGCTTTGGGT	60	3DL
xcfd223	SSR	**F:** AAGAGCTACAATGACCAGCAGA, **R:** GCAGTGTATGTCAGGAGAAGCA	60	3DS
xgwm52	SSR	**F:** CTATGAGGCGGAGGTTGAAG, **R:** TGCGGTGCTCTTCCATTT	60	3DL
xgwm314	SSR	**F:** AGGAGCTCCTCTGTGCCAC, **R:** TTCGGGACTCTCTTCCCTG	55	3DS
xgwm456	SSR	**F:** TCTGAACATTACACAACCCTGA, **R:** TGCTCTCTCTGAACCTGAAGC	55	3DL
xgwm497	SSR	**F:** GTAGTGAAGACAAGGGCATT, **R:** CCGAAAGTTGGGTGATATAC	55	3DS
Xgwm645	SSR	**F:** TGACCGGAAAAGGGCAGA, **R:** GCCCCTGCAGGAGTTTAAGT	55	3DL
S3-113	SCAR	**F:** CGAATTGGATTGGCAGAGGGA, **R:** ACGATCTCCCTACGAATTGCA	60	5Ns
S3-125	SCAR	**F:** GGTGACGAGGGTGTTGGATG, **R:** AGTGAACCGCATGGGTCTTT	58	5Ns
pSc119.2	Oligo-probe	**6-FAM**-CCGTTTTGTGGACTATTACTCACCGCTTTGGGGTCCCATAGCTAT	55	–
pTa535-1	Oligo-probe	**Tamra**-AAAAACTTGACGCACGTCACGTACAAATTGGACAAACTCT TTCGG AGTAT CAGGGTTTC	55	–

**FIGURE 4:**
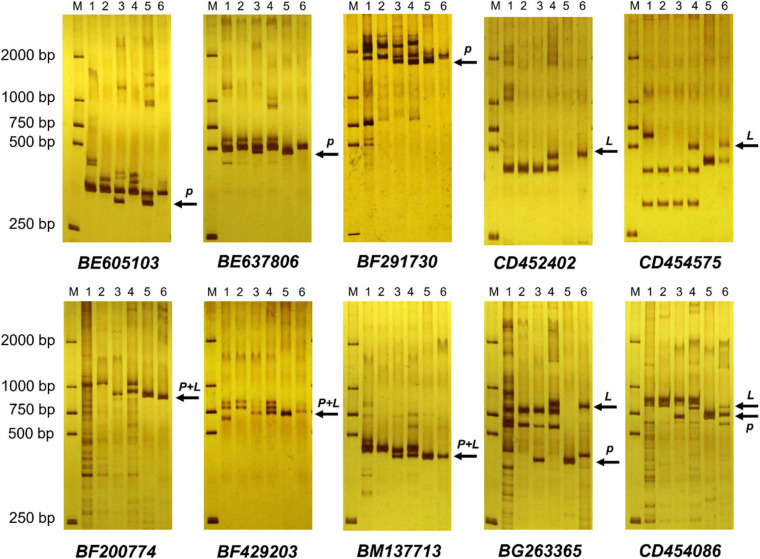
Expressed sequence tag–sequence tagged site (EST-STS) markers analysis of DH109 and DM131. Ten pairs of markers all belonging to the 3rd homoeologous group could amplify Ns genome–specific bands in two derived lines and their alien parents. Among them, marker *BE605103*, *BE637806*, and *BF291730* amplified *P. huashanica* Ns genome–specific bands only in DH109, marker *CD452402* and *CD454575* amplified *L. mollis* Ns genome–specific bands only in DM131 and marker *BF200774*, *BF429203*, *BM137713*, *BG263365*, and *CD454086* amplified Ns genome–specific bands simultaneously in DH109 and DM131. Lane M, DL2000 marker; lane 1, common wheat 7182; lane 2, Trs-372; lane 3, DH109; lane 4, DM131; lane 5, *P. huashanica*; lane 6, *L. mollis*. Arrows indicate the additional Ns genome–specific bands. *P* meant *P. huashanica* Ns genome–specific bands and *L* meant *L. mollis* Ns genome–specific bands.

**FIGURE 5:**
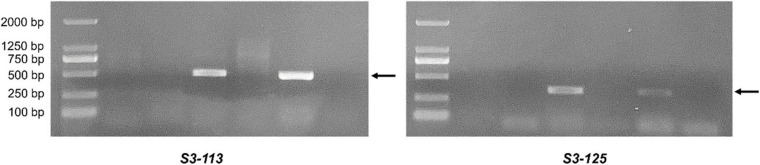
Sequence characterized amplified region (SCAR) markers analysis of DH109 and DM131. SCAR marker *S3-113* and *S3-125* designed base on sequences of *P. huashanica* 3Ns chromosome only amplified bands in DH109 and *P. huashanica* but not in DM131 and *L. mollis.* Lane M, DL2000 marker; lane 1, common wheat 7182; lane 2, Trs-372; lane 3, DH109; lane 4, DM131; lane 5, *P. huashanica*; lane 6, *L. mollis*. Arrows indicate the *P. huashanica* 3Ns chromosome–specific bands.

EST-STS markers and SCAR markers could be used to identify alien chromosomal homoeology in wheat background. We selected 124 pairs of EST-STS markers that distributed among seven homoeologous groups. Ten pairs of STS primers all belonging to the 3rd homoeologous group could amplify Ns genome–specific bands in two derived lines and alien parents, whereas these bands could not be amplified in wheat parents (Figure 4 and Table 1). Among the 10 pairs of markers, three markers were applicative for *P. huashanica* Ns genome, two markers were applicative for *L. mollis* Ns genome, and four markers were universal. The polymerase chain reaction (PCR) results of SCAR markers showed that two *P. huashanica* 3Ns genome–specific SCAR markers, i.e., *S3-113* and *S3-125*, amplified unique and clear bands in DH109 and *P. huashanica* (Figure 5 and Table 1), but not in other materials. These results demonstrated that although 3Ns chromosomes were introduced into both derived lines, DH109 possessed *P. huashanica* 3Ns chromosomes, and DM131 had *L. mollis* 3Ns chromosomes.

Ns genome–specific bands indicated with arrows in Figure 4 amplified in the two lines and their alien parents by EST-STS markers *BF200774*, *BF429203*, and *BM137713* were sequenced. The results showed that DH109 had identical nucleotide sequences with *P. huashanica*, and DM131 was identical to *L. mollis.* Sequences from 3Ns chromosome between *P. huashanica* and *L. mollis* have high similarity. Compared with wheat 21 pairs of chromosomes, these sequences of specific bands from 3Ns chromosomes only matched with the wheat 3rd homoeologous group chromosomes (Table 2).

**TABLE 2:** Analysis of the Ns genome–specific bands amplified in DH109 and DM131 by three EST-STS markers.

Adopted markers	Chromosomal source of products	Size of products (bp)	GenBank accession number	Best match with sequences in wheat chr (percentage)	Alignment of the two genomes
BF200774	*P. huashanica* 3Ns	856	MW114947	3D (87%)	86%
	*L. mollis* 3Ns	864	MW114948	3B (90%)	
BF429203	*P. huashanica* 3Ns	674	MW114949	3A (76%)	74%
	*L. mollis* 3Ns	671	MW114950	3D (83%)	
BM137713	*P. huashanica* 3Ns	464	MW114951	3D (87%)	92%
	*L. mollis* 3Ns	456	MW114952	3D (87%)	

*Each marker amplified similar-size bands in different genomes.*

### FISH and Sequential GISH Analysis of DH109 and DM131

**FIGURE 6:**
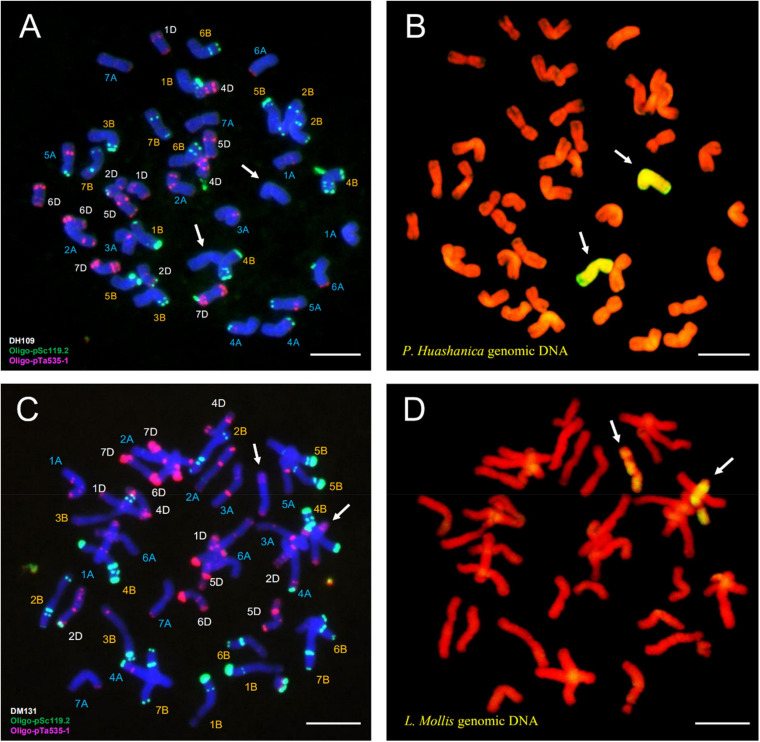
Fluorescence *in situ* hybridization (FISH) and sequential GISH analysis of DH109 and DM131. Oligo-probes pSc119.2 and pTa535-1 were used during metaphase in RTCs to make the chromosomal composition visible. **(A)** FISH karyotype of DH109. **(B)** Two *P. huashanica* chromosomes with yellow–green signals were detected by sequential GISH at the same slide. **(C)** FISH karyotype of DM131. **(D)** Two *L. mollis* chromosomes with yellow–green signals were detected by sequential GISH at the same slide. Chromosomes were counterstained with DAPI (blue) in FISH and PI (red) in GISH. The arrows indicate the introduced alien chromosomes in the two derived lines. Scale bar, 10 μm.

Fluorescence *in situ* hybridization analysis used oligo-primer pSc119.2 and pTa535-1 (Table 1) to distinguish the substituted wheat chromosomes in DH109 and DM131 when compared FISH fluorescent karyotype of the two lines with standard patterns of common wheat Chinese Spring (Annamaria et al., 2003; Tang et al., 2014). In line DH109, a pair of chromosomes with no fluorescent signal replaced wheat 3D chromosomes, which should have red and green signals (Figure 6A, arrows). Sequential GISH analysis was conducted in the slide and indicated that the pair of no-signal chromosomes belonged to *P. huashanica* chromosomes (Figure 6B, arrows). In line DM131, a pair of 3D chromosomes was absent, and another pair of chromosomes with entirely new karyotype appeared in wheat background (Figure 6C, arrows). In sequential GISH experiment, the pair of chromosomes exhibited hybridization signals, which meant they were chromosomes from *L. mollis* (Figure 6D, arrows). Combining with the results of molecular markers, wheat 3D chromosomes were replaced by *P. huashanica* 3Ns chromosomes in DH109 and by *L. mollis* 3Ns chromosomes in DM131. Therefore, line DH109 was a wheat–*P. huashanica* 3Ns (3D) disomic substitution line, and line DM131 was a wheat–*L. mollis* 3Ns (3D) disomic substitution line.

### Wheat 55K SNP Array Analysis of Two Lines

**FIGURE 7:**
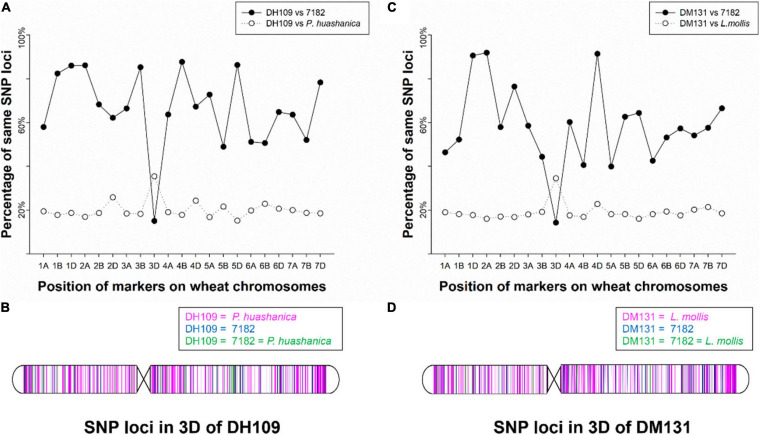
Chromosomal compositions of DH109 and DM131 using wheat 55K SNP array. **(A)** An obvious crossing point in the position of 3D chromosome according to percentages of the same SNP loci between DH109 and its parents. **(B)** Physical positions of the same SNP loci in the 3D chromosome according to genotype of DH109 with its parents. **(C)** An obvious crossing point in the position of 3D chromosome according to percentages of the same SNP loci between DM131 and its parents. **(D)** Physical positions of the same SNP loci in the 3D chromosome according to genotype of DM131 with its parents.

The wheat 55K SNP arrays were used for comparison of fingerprints. When compared with their parents, DH109 exhibited higher similarity with shared wheat parent line 7,182 than DM131 in terms of percentage of same SNP loci in 21-pair chromosomes, and both derived lines showed low similarity with their respective alien parents (Table 3). There was an obvious commonality that cross points were in 3D chromosomes in which two lines had minimum probeset loci allele with wheat parent but had most of the same allele as their respective alien parents at corresponding position (Figures 7A,B). To make comparisons objective, the valid SNPs were arranged in 3D chromosome basing their physical position (Figures 7C,D). The results showed that these SNPs distributed evenly on the entire 3D chromosome, and both derived lines expressed more same alleles in the same positions as their respective alien parents rather than wheat parent 7,182.

**TABLE 3:** Comparison of wheat 55K SNP array data between the two derived lines and their wheat parent 7182.

Chromosome	Number of valid markers	Number of same markers (7182 vs. DH109)	Percentage of same markers (7182 vs. DH109)	Number of same markers (DH109 vs. *P. h.*)	Percentage of same markers (DH109 vs. *P. h.*)	Number of same markers (7182 vs. DM131)	Percentage of same markers (7182 vs. DM131)	Number of same markers (DM131 vs. *L. m.*)	Percentage of same markers (DM131 vs. *L. m.*)
1A	2,624	1,521	58.0%	509	19.4%	1,217	46.4%	497	18.9%
1B	2,595	2,138	82.4%	460	17.7%	1,355	52.2%	470	18.1%
1D	2,138	1,839	86.0%	399	18.7%	1,937	90.6%	378	17.7%
2A	2,622	2,259	86.2%	442	16.9%	2,410	91.9%	419	16.0%
2B	2,600	1,775	68.3%	484	18.6%	1,506	57.9%	442	17.0%
2D	2,247	1,397	62.2%	580	25.8%	1,718	76.5%	377	16.8%
3A	2,174	1,444	66.4%	400	18.4%	1,273	58.6%	391	18.0%
3B	2,595	2,213	85.3%	473	18.2%	1,150	44.3%	496	19.1%
3D	1,693	254	15.0%	599	35.4%	241	14.2%	584	34.5%
4A	2,592	1,650	63.7%	493	19.0%	1,561	60.2%	455	17.6%
4B	2,556	2,242	87.7%	454	17.8%	1,036	40.5%	430	16.8%
4D	1,420	955	67.3%	345	24.3%	1,298	91.4%	323	22.7%
5A	2,611	1,900	72.8%	438	16.8%	1,041	39.9%	473	18.1%
5B	2,586	1,266	49.0%	558	21.6%	1,620	62.6%	468	18.1%
5D	1,737	1,499	86.3%	263	15.1%	1,118	64.4%	278	16.0%
6A	2,623	1,342	51.2%	518	19.7%	1,115	42.5%	475	18.1%
6B	2,547	1,290	50.6%	582	22.9%	1,354	53.2%	492	19.3%
6D	1,728	1,120	64.8%	356	20.6%	990	57.3%	303	17.5%
7A	2,579	1,640	63.6%	515	20.0%	1,396	54.1%	520	20.2%
7B	2,487	1,294	52.0%	465	18.7%	1,432	57.6%	531	21.4%
7D	2,305	1,806	78.4%	426	18.5%	1,533	66.5%	426	18.5%
A genome	17,825	11,756	66.0%	3,315	18.6%	10,013	56.2%	3,230	18.1%
B genome	17,966	12,218	68.0%	3,476	19.3%	9,453	52.6%	3,329	18.5%
D genome	13,268	8,870	66.9%	2,968	22.4%	8,835	66.6%	2,669	20.1%
Total	49,059	32,844	66.9%	9,759	19.9%	28,301	57.7%	9,228	6.7%

### Differences in Diseases Resistance and Agronomic Traits of DH109 and DM131

**FIGURE 8:**
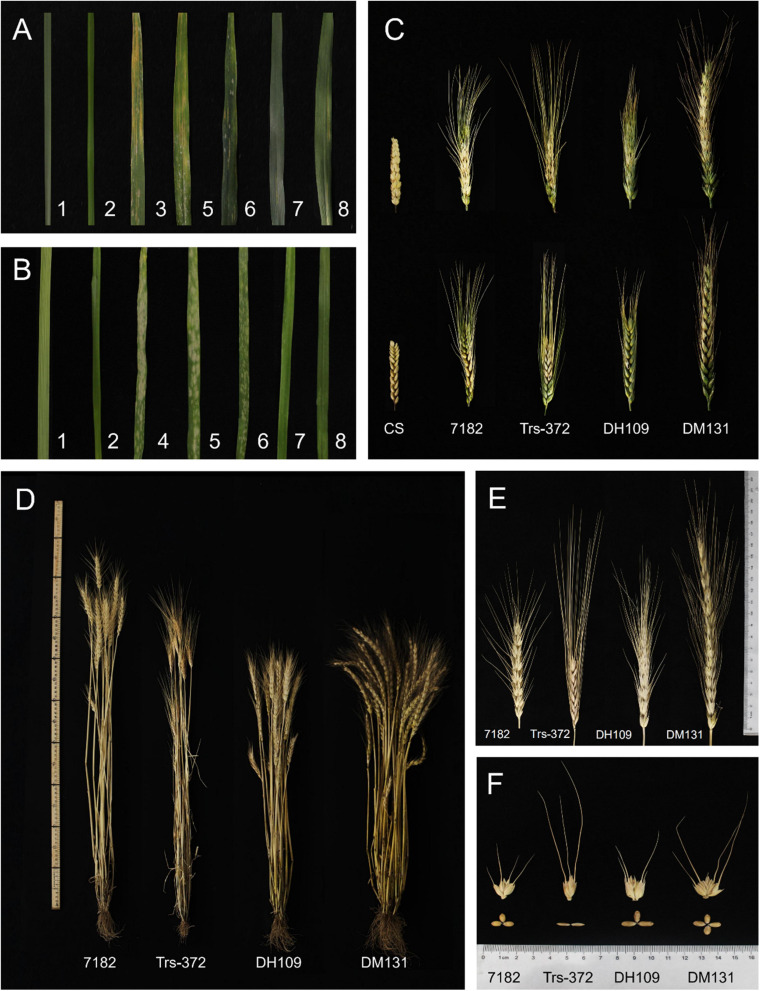
Diseases resistance and agronomic traits of DH109 and DM131, their wheat parents, and controls. **(A)** Symptoms in response to inoculation with a mixture of *Pst* races in the adult stage. **(B)** Symptoms in response to inoculation with a mixture of *Bgt* isolate E09 in the seeding stage. **(C)** Spike symptoms after injection with *Fusarium graminearum*. **(D)** Plants of materials. **(E)** kernels of materials. **(F)** Spikes of materials. The materials in the Figure are 1, *L. mollis*; 2, *P. huashanica*; 3, control Mingxian 169; 4, control Huixianhong; 5, common wheat 7182; 6, *durum* wheat Trs-372; 7, line DH109; 8, line DM131.

The response of materials to wheat stripe rust at adult stage was tested in the field and all materials grown under the same condition to ensure the accuracy of results. The ITs of the seven materials were as follows: susceptible control Mingxian 169 (IT = 4), line 7,182 (IT = 3), Trs-372 (IT = 2), DH109 (IT = 1), DM131 (IT = 2), *L. mollis* (IT = 0), and *P. huashanica* (IT = 0) (Figure 8A). This indicated that the introduced alien chromosomes did not make the two lines exhibit great resistance to mixed *Pst* races (CYR32, 33, 34).

Resistance to powdery mildew was evaluated in growth chamber in the seedling age and infected using *Bst* isolate E09. The ITs of seven materials were as follows: susceptible control Huixianhong (IT = 4), line 7182 (IT = 3+), Trs-372 (IT = 3), DH109 (IT = 0), DM131 (IT = 0), *L. mollis* (IT = 0), and *P. huashanica* (IT = 0) (Figure 8B). It is obvious that DH109 and DM131 were almost immune to inoculated *Bst* isolate, indicating that both lines acquired powdery mildew resistance genes from their alien parent.

The spikelets that kraurotic or covered with mycelium were considered infected after injection. Among them, only DH109 expressed high FHB resistance (RI = 1.57, ISR = 9.86%); susceptible control and other materials all had severe symptoms: CS (RI = 4.87, ISR = 93.87%), 7182 (RI = 4.4, ISR = 70.58%), Trs-372 (RI = 4.64, ISR = 76.74%), and DM131 (RI = 4.37, ISR = 80.25%) (Figure 8C). In response to FHB strain PH1, DH109 and DM131 expressed visible difference that DH109 was superior to DM131.

The morphological traits of the two substitution lines and their wheat parents (7182 and Trs-372) could be seen in Figures 8D–F and Table 4. DH109 and DM131 both exhibited shorter plant compared with their wheat parents 7182 and Trs-372, but DH109 had bigger kernels, and DM131 had longer spikes, more kernels per spike, and tiller number (at *p* = 0.05 and *p* = 0.01). However, grain quality indicators showed that the two lines fell in between common wheat 7182 and durum wheat Trs-372, meaning grain quality of DH109 and DM131 had no significant improvement (Table 5).

**TABLE 4:** Morphological traits of common wheat 7182, durum wheat Trs-372, DH109, and DM131.

Material	Plant height (cm)	Tiller number	Spike length (cm)	Spikelets per spike	Kernels per spike	Thousand kernel weight (g)
7182	79.9 ± 3.3Aa	12 ± 2BCbc	9.04 ± 0.59Bb	18 ± 2Bb	55 ± 4Bb	37.78 ± 0.82Bb
Trs-372	70.7 ± 2.6Bb	11 ± 2Cc	6.87 ± 0.4Cc	14 ± 3Cc	39 ± 3Cc	32.18 ± 0.94Cc
DH109	62.3 ± 2.1Cd	14 ± 2Bb	8.5 ± 0.62Bb	17 ± 2BCb	55 ± 3Bb	43.66 ± 0.61Aa
DM131	65.7 ± 1.9Cc	28 ± 2Aa	16.46 ± 1.01Aa	24 ± 3Aa	71 ± 3Aa	38.25 ± 0.82Bb

*Different uppercase and lowercase letters indicate significant differences at p < 0.01 and p < 0.05, respectively, between the two substitution lines and their parents.*

**TABLE 5:** Grain quality results of DH109, DM131, and their wheat parents.

Material	Crude protein content	Gluten protein content	Starch content	Subsidence value	Volume–weight	Dough stability time	Flour field
7182	13.04 ± 0.26Cc	25.33 ± 1.01Cc	56.49 ± 0.765Bb	24.41 ± 1.13Cc	788.5 ± 6Aa	2.2 ± 0.7Bc	72 ± 2ABb
Trs-372	16.67 ± 0.45Aa	34.44 ± 0.99Aa	63.79 ± 1.42Aa	41.19 ± 1.07Aa	786 ± 2Aa	6.8 ± 1.6Aa	76 ± 2Aa
DH109	14.47 ± 0.32Bb	34.25 ± 0.57Aa	62.73 ± 0.98Aa	34.85 ± 1.53Bb	754.5 ± 4Bb	4.9 ± 0.3Ab	66 ± 1Cd
DM131	14.3 ± 0.54BCb	30.47 ± 0.61Bb	58.36 ± 0.91Bb	24.695 ± 1.77Cc	757 ± 2Bb	1.4 ± 0.7Bc	68.5 ± 1BCc

*Different uppercase and lowercase letters indicate significant differences at p < 0.01 and p < 0.05, respectively, between the two substitution lines and their parents.*

## Discussion

The most common way to utilize the genome of relative species of wheat is to create wheat-alien–derived lines, including addition lines, substitution lines, translocation lines, and introgression lines, and then cross plus multiple backcross with wheat varieties to obtain new wheat germplasms containing objective traits of alien species (Li et al., 2008). The Ns genome consists of seven homoeologous groups (1–7Ns) that all have been testified useful for wheat breeding improvement because of many beneficial genes: leaf rust resistance genes located in 1Ns, 3Ns, and 7Ns (Du et al., 2012, 2014c; Pang et al., 2014); stripe rust resistance genes located in 2Ns, 3Ns, 4Ns, 5Ns, and 7Ns (Du et al., 2012, 2014a, 2014b, 2014c; Li J. C. et al., 2019; Li et al., 2020a); powdery mildew resistance genes located in 1Ns and 5Ns (Han et al., 2020; Li et al., 2020b); spike characters–related genes located in 4Ns and 6Ns (Du et al., 2013, 2014a); and gluten synthesis–related genes located in 1Ns, 5Ns, and 6Ns (Zhao et al., 2010; Du et al., 2013; Li J. C. et al., 2019). Years of research and numerous evidences, i.e., chromosomal pairing in meiotic stage, molecular markers studies, southern hybridization, and GISH analysis, all show that *Leymus* share the same Ns genome from *Psathyrostachys*, whereas Ns genome in *Leymus* ought to be a mutational version (Wang et al., 2006; Yen et al., 2009). In the long course of evolution, variation in genetic material is inevitably large, which makes species from different genera appear to be closer than they are within the same genus in the cluster analysis of the *Psathyrostachys*–*Leymus* group according to the results of restriction fragment length polymorphism (Anamthawat-Jonsson and Bodvarsdottir, 2001). In this case, are there big differences in agronomic traits if Ns genome chromosomes from the same homoeology but different genera are separately introduced to wheat background? Therefore, wheat alien–derived lines with the same homoeologous chromosomes were created for such comparison. In the current study, we developed and identified two novel wheat–alien substitution lines, which one carried a pair of *P. huashanica* 3Ns chromosomes and one carried a pair of *L. mollis* 3Ns chromosomes. Both pairs of the Ns chromosomes are stable and inheritable and caused obvious changes of their wheat receptor parent in agronomic traits and disease resistance.

Classical cytogenetics is a necessary way to give an insight into chromosomal composition and transmission of materials. When wheat-alien–derived lines have consistent traits in several successive years, the compositions and behaviors in their RTCs and PMCs need to be observed (Cifuentes and Benavente, 2009). In this study, observations showed that DH109 and DM131 both had 42 chromosomes in somatic cells, and they could pair up to form 21 bivalents in meiotic metaphase I. Subsequently, half of the chromosomes, respectively, moved to cell pole without lagging in anaphase I. GISH technology was first applied to identify alien chromosome(s) in wheat in 1989 and improved by Le et al. (1989) and Mukai and Gill (1991). Since then, the content and behavior of alien chromosome in wheat background could be visualized. GISH analysis suggested that DH109 had 40 wheat chromosomes plus two *P. huashanica* chromosomes, and the two chromosomes were from one homoeology because of their behaviors in pairing, segregation, and transmitting. Same as in DH109, two *L. mollis* chromosomes in DM131 were from one homoeology and stably inherited.

We only knew that a pair of wheat chromosomes was substituted by a pair of alien chromosomes in the two lines through GISH experiments; therefore, genome-specific molecular markers were adopted to determine homoeology of these alien chromosomes and the lost wheat chromosomes. A wheat–*L. mollis* 2Ns, 3Ns (2D, 3D) double substitution line and a wheat–*P. huashanica* 5Ns (5D) substitution line were identified by Li J. C. et al. (2019) and Zhao et al. (2019) using SSR, EST-STS, and SCAR markers. SSR markers located at a specific position of wheat chromosome could be regarded as tags for the presence of chromosomes (arms) (Röder et al., 1998). EST-STS markers were designed from coding DNA and were generally highly conserved, so they could be used for comparative genomic studies between distant species (Kamaluddin et al., 2017). In this study, we found that SSRs on 3D chromosomes amplified D genome-specific bands in wheat parent 7182, but not in lines DH109 and DM131. EST-STSs on the third homoeologous group amplified Ns genome-specific bands in DH109, DM131, and alien species. SCARs were designed based on sequences of *P. huashanica*–amplified bands only in materials containing *P. huashanica* 3Ns chromosomes. So, it could be preliminarily determined that DH109 was a wheat–*P. huashanica* 3Ns (3D) substitution line, and DM131 was a wheat–*L. mollis* 3Ns (3D) substitution line. It was worth noticing that although EST-STSs and SCARs from third homoeologous group could be used to identify 3Ns chromosomes, the target bands might be different in different species, e.g., *P. huashanica* and *L. mollis*, which showed the same homoeologous chromosomes containing different genetic materials in different genera. Parallel bands sequencing results indicated that 3rd homoeologous group chromosomes from the three genera had high homoeology, which mainly showed up as dispersedly multiple-bases differences rather than continuous differences in long segments.

**FIGURE 9:**
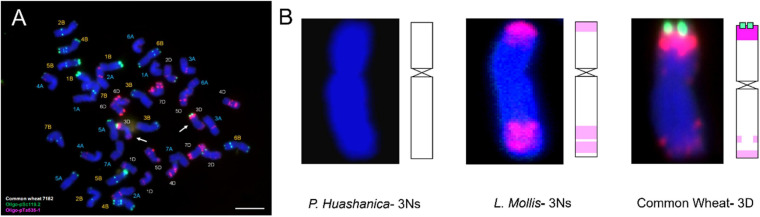
FISH karyotype of third homoeologous chromosomes from common wheat 7182, *P. huashanica*, and *L. mollis.* **(A)** FISH karyotype of common wheat 7182 during metaphase and the arrows indicate the wheat 3D chromosomes. **(B)** FISH karyotypes and idiograms of wheat 3D, *P. huashanica* 3Ns, and *L. mollis* 3Ns chromosomes. Gradation of color indicates the intensity of the fluorescence signal. Scale bar, 10 μm.

Fluorescence *in situ* hybridization analysis was an efficient and reliable way to detect structural rearrangements and replacements of wheat chromosomes because appropriate match of FISH oligo-probers could distinguish 21 pairs of wheat chromosomes according to the standard FISH karyotypes (Huang et al., 2018). The most commonly used FISH probers were oligo-GAA (A and B genome), oligo-pSc119.2 (B genome), oligo-pTa535 (A and D genome), and oligo-pAs1 (A and D genome) (Danilova et al., 2012; Tang et al., 2014). In today’s study, matching of pSc119.2 and pTa535 was employed, and the results showed that DH109 and DM131 both lost their 3D chromosomes but possessed a pair of chromosomes whose FISH karyotypes were brand new. Therefore, sequential GISH was conducted to further characterize these exceptional chromosomes in the same slide. With the results of molecular markers, the chromosomes with novel karyotypes in DH109 were *P. huashanica* 3Ns chromosomes and in DM131 were *L. mollis* 3Ns chromosomes. Comparing oligo-probe pSc119.2 and pTa535-1 FISH pattern and ideogram of third homoeologous chromosomes from different genera, big differences could be seen in Figure 9. It was clear that the terminal part of chromosome arms exhibited red and green fluorescent signals in wheat 3D chromosome and red fluorescent signals in *L. mollis* 3Ns chromosome. However, none of the signals were in *P. huashanica* 3Ns chromosomes. Repetitive sequences have been estimated to be between 16 and 45% in cereal genome, which are helpful in differentiating closely related species, detecting interspecific hybrids and introgressions (Anamthawat-Jónsson and Heslop-Harrison, 1993). Therefore, the results demonstrated that the relationship between *L. mollis* and *Triticum* was closer than *P. huashanica*, and *L. mollis* had distant phylogenetic relationship with *P. huashanica*, which supported the inferences (i.e., donor species of Ns genome to *Leymus* was not *P. huashanica*) of Bodvarsdottir and Anamthawat-Jonsson (2003) and Wang et al. (2006) based on their studies of SSR markers and Southern blot.

Molecular marker-assisted selection has three main categories comprising markers based on molecular hybridization, e.g., restriction fragment length polymorphism and variable number of tandem repeat; markers based on PCR technology, e.g., random amplified polymorphism DNA and SSR; and markers based on high-throughput DNA sequence, e.g., SNP arrays and specific-locus amplified fragment sequencing (SLAF-seq) (He et al., 2003; Varshney et al., 2005; Kamaluddin et al., 2017). Among them, SNP arrays are likely to be the most important tool in gene mapping and study the relationship of species (Zhou et al., 2018; Zhao et al., 2019). Wheat SNP arrays were first applied in identification of wheat-alien–derived line by Li J. C. et al. (2019) and Li et al. (2020b) and successfully verified two derived lines. In the present study, we compared the genotype of DH109 with its parents and DM131 with its parents in each locus of wheat 55K SNPs that spread on 21 chromosomes, respectively. It was consistent with results from molecular markers and FISH analysis, which showed DH109 was a wheat–*P. huashanica* 3Ns (3D) substitution line and DM131 was a wheat–*L. mollis* 3Ns (3D) substitution line. Compared with the results of previous articles of 15K SNP array in this section, it can be obviously found that low-density SNP array got higher resolution in substitution-occurred or translocation-occurred homoeologous group. Therefore, in the identification of exogenous substances, it might be more efficient and cheaper to use 15K or 35K SNP arrays rather than 90K or 660K SNP arrays.

The introduction and replacement of alien chromosome(s) or segment(s), except for B-chromosomes, usually cause changes in the traits of recipient plant (Camacho et al., 2000; Jones et al., 2008). In wheat, these changes might be obvious in morphologic traits, such as plant height (Wang S. W. et al., 2019) and kernel size (Zhang et al., 2016); they also might be invisible in resistance or grain quality (He et al., 2017; Li X.Y. et al., 2019). Unfortunately, not all alien chromosomes were beneficial to wheat because they might result in worse agronomic traits, e.g., small spike and less tiller (Wang J. et al., 2019), and decreased processing quality (Liu et al., 2004). Therefore, return to breeding requirement, the most important criterion to access value of one wheat-alien–derived line, was its agronomic trait. In this study, two substitution lines DH109 and DM131 both expressed high resistance to powdery mildew in their seeding age. Moreover, DH109 also had high FHB resistance and bigger kernels, and DM131 had longer spike and more tiller number, which were outstanding agronomic traits for wheat improvement. Although the two derived lines both possessed a pair of alien chromosomes that belonged to the 3rd homoeology and named 3Ns, they had obviously different agronomic traits because DH109 had *P. huashanica* 3Ns chromosomes, and DM131 had *L. mollis* 3Ns chromosomes. The chromosomal recombination and crossing with durum wheat Trs-372 might cause individual difference even in the same generation; for example, DH109 was more like common wheat 7182 in plant type, and DM131 was more like durum wheat Trs-372 in grain quality. However, enhanced/increased disease resistance and some excellent traits are most likely caused by introduction of alien chromosomes. Relatively large differences in agronomic traits between DH109 and DM131 supported that there were many different genes in Ns genome between *P. huashanica* and *L. mollis*.

## Conclusion

In this study, a novel wheat–*P. huashanica* disomic substitution line named DH109 and a novel wheat–*L. mollis* disomic substitution line named DM131 were identified by using molecular and cytogenetic methods. Although both two lines were developed because of the substitution of exogenetic 3Ns chromosomes and wheat 3D chromosomes, they were obviously different in bands of molecular markers, FISH karyotype and agronomic traits. Thus, Ns genome from *P. huashanica* and *L. mollis* had big differences. Furthermore, after multiple generation advancement, the two lines have been stable in morphology and genetics. Line DH109 expressed superior resistance to powdery mildew and FHB, and line DM131 had powdery mildew resistance, longer spike, and more tiller number. Therefore, the two lines could have different preferences toward wheat breeding and Ns genome research.

## Data Availability Statement

The datasets generated for this study can be found in online repositories. The names of the repository/repositories and accession number(s) can be found in the article/Supplementary Material.

## Author Contributions

JCL and JJL conducted the experiments. LZ and XNC analyzed the data. JW and QY contributed new reagents and analytical tools. ZY contributed new methods. JZ and XHC conceived and designed the research. JCL wrote the manuscript. All authors read and approved the final version of the manuscript.

## Conflict of Interest

The authors declare that the research was conducted in the absence of any commercial or financial relationships that could be construed as a potential conflict of interest.

## Footnotes

1 https://wheat.pw.usda.gov/SNP/new/pcr_primers.shtml

2 https://blast.ncbi.nlm.nih.gov/Blast.cgi

3 https://urgi.versailles.inrae.fr/blast/
